# Integrated IMU with Faster R-CNN Aided Visual Measurements from IP Cameras for Indoor Positioning

**DOI:** 10.3390/s18093134

**Published:** 2018-09-17

**Authors:** Lin Zhang, Taoyun Zhou, Baowang Lian

**Affiliations:** Department of Information and Communication, School of Electronics and Information, Northwestern Polytechnical University, Xi’an 710129, China; taoyun_2000@mail.nwpu.edu.cn (T.Z.); bwlian@nwpu.edu.cn (B.L.)

**Keywords:** IMU, object detection, vision measuring, EKF, Faster R-CNN

## Abstract

Considering the radio-based indoor positioning system pertaining to signal degradation due to the environmental factors, and rising popularity of IP (Internet Protocol) cameras in cities, a novel fusion of inertial measurement units (IMUs) with external IP cameras to determine the positions of moving users in indoor environments is presented. This approach uses a fine-tuned Faster R-CNN (Region Convolutional Neural Network) to detect users in images captured by cameras, and acquires visual measurements including ranges and angles of users with respect to the cameras based on the proposed monocular vision relatively measuring (MVRM) method. The results are determined by integrating the positions predicted by each user’s inertial measurement unit (IMU) and visual measurements using an EKF (Extended Kalman Filter). The results experimentally show that the ranging accuracy is affected by both the detected bounding box’s by Faster R-CNN height errors and diverse measuring distances, however, the heading accuracy is solely interfered with bounding box’s horizontal biases. The indoor obstacles including stationary obstacles and a pedestrian in our tests more significantly decrease the accuracy of ranging than that of heading, and the effect of a pedestrian on the heading errors is greater than stationary obstacles on that. We implemented a positioning test for a single user and an external camera in five indoor scenarios to evaluate the performance. The robust fused IMU/MVRM solution significantly decreases the positioning errors and shows better performance in dense multipath scenarios compared with the pure MVRM solution and ultra-wideband (UWB) solution.

## 1. Introduction

Indoor positioning technologies [1,2,3] are necessary and technical prerequisites for various industrial and consumer applications in location-based services. This location solution typically provides a user with a reliable and accurate pose estimation of a device or a person in public and private areas [4,5,6,7], like an airport, hotel, mall, home, etc. However, as GNSS (Global Navigation Satellite System) signal is not continually available in indoor environments and the presence of unavoidable issues in complex indoor areas, such as multipath and non-line of sight, the high precision and reliable indoor positioning is not easy to be achieved in realistic conditions. Based on these constraints, developing a low cost, reliable, and infrastructure-free or infrastructure-less positioning solution for consumers remains an open challenge at present.

To address this problem, much of the recent research focuses on sensor-based indoor positioning technologies. The representative solutions determining locations in indoor environments comprise Wi-Fi [8], Bluetooth [9], iBeacon [10], RFID [11,12], infrared [13], inertial sensors [14], magnetometer [15], ultra-wideband (UWB) [16,17], etc. Wi-Fi technology draws increasing attention among above solutions due to its popularization in cities. However, the susceptible wireless makes the positioning of the received signal strength indicator (RSSI)-based [18,19] method getting worse when multipath effect occurs in dense urban areas. To further improve the accuracy, building a specialized fingerprint [20,21] for Wi-Fi signal in advance is implemented, but it requires periodic renewal to rebuild the database. Recently, UWB has been widely used due to its characteristics, such as good penetrability, high precision, and anti-multipath ability. Nevertheless, the UWB systems unavoidably suffer from interference caused by narrow wide signal from coexisting systems and non-line of sight (NLOS) conditions [22] restrict the performance substantially. Dead Reckoning (DR) [23,24] utilizes a gyroscope and accelerometer to infer movements of a pedestrian based on measurements and previous locations. However, inertial sensors suffer from biases and drift errors that will accumulate over time. Therefore, with the limits of cost, size, performance, production, etc., the hybrid positioning technologies with inertial sensors are introduced to efficiently reduce the drift errors and further improve positioning accuracy, such as PDR/Bluetooth [25], INS/Wi-Fi [26], etc. The optimization-based INS/UWB approach in reference [27] achieved RMSE of position and orientation approximately as 3-cm and less than $1^{{}^{\circ}}$ using an IMU and a UWB transmitter placed on a body and 10 UWB receivers deployed in a test room. However, this fusion system requires a more accurate distribution model to remove large amounts of the time of arrival (TOA) measurements outliers caused by multipath and NLOS conditions and combines the corrected UWB measurements with inertial measurements to determine six degrees-of-freedom (DOF) pose of the moving body.

Compared with the conventional radio-based positioning methods, vision-based positioning [28,29] is proved to become a greatly promising navigation and positioning technique in various applications. Visual simultaneous localization and mapping (SLAM) [30,31] has been extensively applied in various fields, such as virtual reality/augmented reality, robotic mapping and navigation, autonomous vehicle, etc. Feature detection and description, graph optimization and loop closure are key techniques to implement SLAM algorithms significantly. In contrast with binocular or stereo vision, monocular vision methods remain a challenge to provide robust and accurate pose estimations with good performances due to lacking scale factor and depth information. The integrated method with inertial sensors is able to overcome these limitations of monocular vision-only and IMU-only positioning by using their complementary properties. Fast movement information in short period can be predicted by IMU, and drift errors from IMU can be corrected by vision measuring effectively. A popular representative, visual inertial odometry (VIO) [32,33], which has great potential of resolving the estimation problems in these above applications. Popular VIO algorithms include the filter-based MSCKF [34] and optimization-based Okvis [35]. Recently, a robust and versatile monocular visual-inertial state estimator, i.e., VINS-Mono, comprises optimization-based VIO, online loop detection, tightly-coupled re-localization and four DOF pose graph optimization in reference [36]. The experiments show superior performance by comparing against Okvis by running on mobile devices. In [37] with the use of urban 3D model, the position and orientation of camera relative to the known street object is estimated by using efficient PnP algorithm. The final average position for the fusion of IMU with the camera data is 0.22 m in experiment conditions. This method largely relies on the rich and reliable 3D model to determine absolutely position especially in a low visibility area.

In practical application, indoor tracking and locating of a moving human target at a low cost with good performance remains an open issue. The popular vision-based methods to deal with identifying a pedestrian in images in various environments can be categorized into either traditional feature detector or deep learning-based methods. In [38], pedestrians are recognized through the use of algorithms based on edge density and symmetry maps. However, the position error depends on images sequences categories with mean location error at 0.98 m in backwards running. In [39], a Bayesian-based vision tracking system providing user’s position estimation made the RMSE for position improve to 20-cm by integrating with inertial sensors through an EKF. In the recent years, deep learning [40] has made significant breakthroughs in visual recognition, speech recognition, and natural language processing. In addition, positioning approach with assistant of deep learning is becoming an active research area. In paper [41], a novel indoor positioning and tracking system fusing magnetic signal and visual images with a deep convolutional neural network (CNN) to extract deep features for measurements was presented. The main contribution of this paper [42] is to leverage CNN to build a proper radio propagation model which is applied in crowed scenarios. Reference [43] discussed a state-of-art survey on pedestrian detection and tracking methods by utilizing computer vision and deep learning techniques. In [44], the authors used CNN to classify pedestrian in images and showed higher levels of accuracy compared with traditional SVM approach with Haar features. The presented work above provides a new perspective for developing and resolving the indoor positioning and tracking issues.

The rise and rapid progress of 5G and internet of things technologies [45,46,47] allow diverse wireless devices to be connected by larger-scale wireless sensor networks [48] for exchanging and sharing information, and they have been widely used in medical treatment, smart home, higher education, intelligent transportation [49,50,51], etc. This technology commonly relies on measurements information among every pair of nodes, like ranges and angles, to realize relative or absolute localization based on wireless sensors. Reference [52] proposed an approach combining IMU and UWB ranging measurements for a relative positioning among multi users with a particle filter. These range-based and angle-based localization methods obtaining peer to peer measurements can be implemented by using cameras instead of wireless sensors.

In consequence, we propose a novel indoor positioning approach combining IMU and Faster R-CNN-aided relative measurements from IP cameras to determine pose estimations of users, which is inspired by the rising popularity of IP cameras in cities and the complementary properties of IMU with cameras. This solution leverages an extended Kalman filter to tightly fuse IMU data and relative ranges and angles with respect to cameras obtaining by our presented monocular vision relatively measuring (MVRM) method. We conducted this approach in indoor environments and evaluated the performances of the proposed approach in stationary objects and pedestrian blockage scenarios. The experiment results show that the proposed approach can significantly reduce positioning errors and enhance reliability during blockage period.

The rest of this paper is structured as follows. In Section 2, a concept of the proposed integrated system is presented. In Section 3, Faster R-CNN based object detection is analyzed briefly, and the proposed MVRM method is modelled. This section also formulates the integrated IMU/MVRM for a group of users and cameras. In Section 4, the experiment setup and the results of the proposed approach are introduced in real indoor environments. In Section 5, the conclusion and further work are summarized.

## 2. System Overview

**Figure 1:**
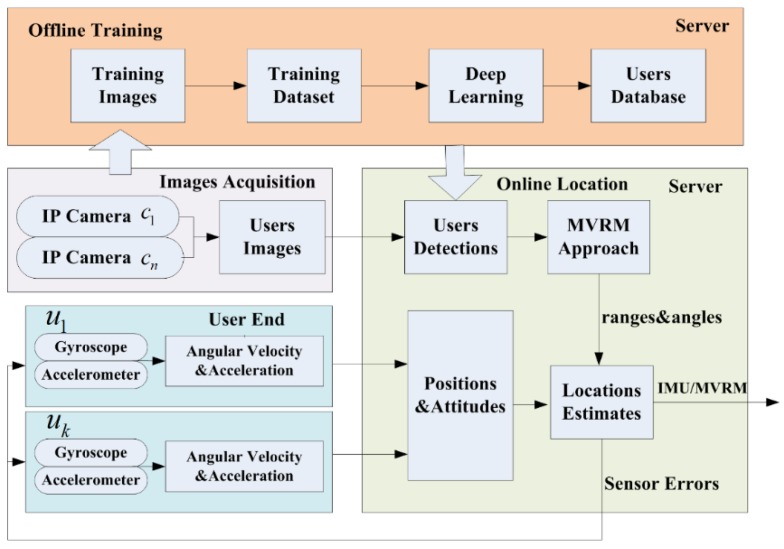
The concept of the proposed inertial measurement unit (IMU)/monocular vision relatively measuring (MVRM) integrated system.

The concept of the proposed IMU/MVRM integrated system for indoor positioning comprising two phases: offline training and online location, is shown in Figure 1. In the offline phase, IP cameras capture images of users, and send them to a server. These uploaded images are used to create a training dataset for training a model of detecting multi-users based on a deep neural network. In the online phase, cameras start to take an image of a user in real time when a positioning request is sent by this user and send it to the server to detect the user in this image with the trained model. Meanwhile, the user end sends the pose estimations predicted by its own IMU to the server. The fusion filter fuses IMU’s predictions with the ranges and angles of this user with respect to these cameras obtained by the proposed MVRM method. Finally, the corrected estimations and sensor biases will be resent to the user end.

## 3. Methods

### 3.1. Faster R-CNN Based Object Detection

In the last few years, object detection by using deep learning has attracted a great deal of attention, in particular, using the typical region with CNN (R-CNN) [53]. This technology utilizes CNNs to extract features from all candidates which makes it become a time-consuming work due to its high computational cost during training and test periods. To speed-up, Fast R-CNN [54] and Faster R-CNN [55] are introduced consecutively. Faster R-CNN with a region proposal network (RPN) specialized in proposals generation merges region proposals, features extraction, classification and bounding box regression into just one deep neural network which significantly increases the running speed by 250 times, as opposed as R-CNN. In this part, Zeiler and Fergus net (ZF) [56] based Faster R-CNN is used to detect users in images.

**Figure 2:**
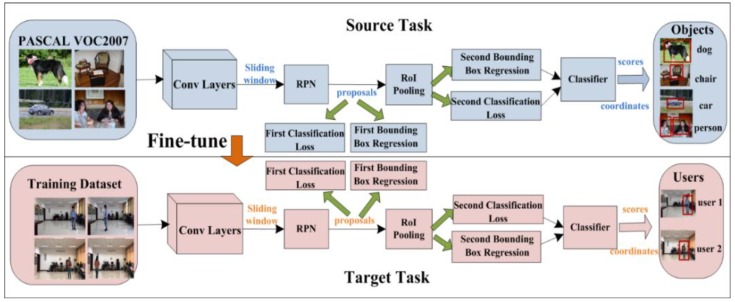
Schematic view of fine-tuning Faster R-CNN (Region Convolutional Neural Network).

The performance of a deep learning partially depends on the size of a dataset. However, it is indeed hard to build a large-scale dataset for a particular detection task, like our multi-users detection. Therefore, we adopt the pre-trained Faster R-CNN, a 20 general class objects detection model on PACSAL VOC 2007 detection benchmarks [57] in source task, to be fine-tuned on our training dataset in target task, which efficiently improves the training performance and enhances generalization by reducing overfitting. Flowchart of fine-tuning Fast R-CNN is depicted on Figure 2. As shown, the model parameters can be optimized by fine-turning on the training data, and the output of the target task is the optimized model of multi-users detection which can accurately identify and locate users (trained pedestrians in offline) in images.

Generally, the performance of object detection can be evaluated in two aspects: mean average precision (mAP) and intersection over union (*IoU*). In this paper, mAP defined in PASCALVOC 2012 [58] is used to evaluate the fine-tuned Faster R-CNN.

*AP* represents as an area under the Precision-Recall curve:

(1) AP=∫Pd(R),

where *P*, *R* indicate the precision and recall rate of detection, written as

(2) $$P=\frac{Tp}{Tp+Fp},$$

(3) $$R=\frac{Tp}{Tp+Fn},$$

where Tp, Fp mean the number of true and false positive samples, Fn means the number of false negative samples.

*IoU* is used to evaluate the performance of location which measures the overlapping ratio of the bounding boxes between predication and truth.

(4) $$IoU=\frac{F\cap G}{F\cup G},$$

where *F* denotes the area of bounding box predicated by the fine-tuned Faster R-CNN, *G* denotes the actual area of bounding box.

### 3.2. Monocular Vision-Based Relatively Measuring Method

As mentioned previously, the proposed monocular vision-based relatively measuring method utilizes the locations in pixels of detected users in images with the use of the fine-tuned Faster R-CNN and the real height in meters of the users in word coordinate system to estimate ranges and angles of users with respect to cameras based on the ranging and angulation model presented in this section. Unlike radio-based TOA, angle of arrival (AOA), received signal strength indicator (RSSI), etc., the measuring accuracy of this vision-based method will not decrease due to multipath interference.

#### 3.2.1. Ranging Model

**Figure 3:**
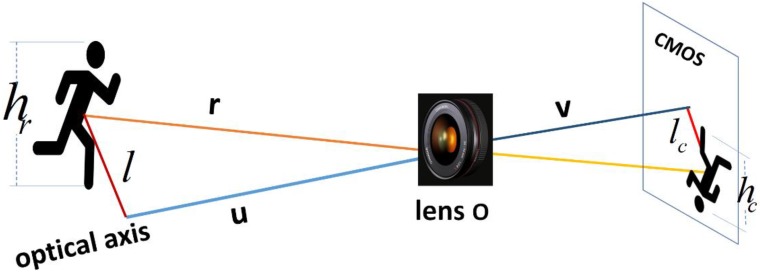
The monocular vision-based ranging model.

In general, users being detected are not located on the optical axis of a camera. The relative range of a user respect to a camera is the distance between the user and optical center of the lens. As shown in Figure 3, r is the relative range, o is optical center of the lens, u, v are object distance and image distance respectively, l is the distance from a user to the optical axis, $l_{c}$ is the distance from image of the user to the center of the film measured on COMS sensor, $h_{r}$ denotes real height of the user, $h_{c}$ denotes image height of the user measured on CMOS sensor, the relationship of $h_{r}$ and $h_{c}$ is shown as:

(5) $$\frac{h_{r}}{h_{c}}=\frac{u}{v},$$

where $h_{c}$ is expressed as:

(6) $$h_{c}=\frac{n_{v}}{re},$$

(7) $$re=\frac{\sqrt{h_{p}^{2}+v_{p}^{2}}\cdot f_{35mm}}{diag_{35mm}\cdot f},$$

where $n_{v}$ is user height in pixels, re is the number of count of pixels per unit, $f_{35mm}$ and f are 35 mm equivalent focal length and focal length in word units respectively, $h_{p}$ and $v_{p}$ are image dimensions, $diag_{35mm}$ is length of the diagonal of 35 mm film.

Similarly, the relationship of l and $l_{c}$ is expressed as:

(8) $$l=l_{c}\cdot \frac{h_{r}\cdot re}{n_{v}},$$

(9) $$l_{c}=\sqrt{{\left(\frac{d_{h}}{re}\right)}^{2}+{\left(\frac{d_{v}}{re}\right)}^{2}},$$

where $d_{h}$ and $d_{v}$ are distances from image of a user to the center of the film measured in pixels in horizontal and vertical directions, respectively.

The estimated relative range r is given by:

(10) $$r=\frac{h_{r}\cdot \sqrt{l_{c}^{2}+v^{2}}}{h_{c}}=\frac{h_{r}\cdot re}{n_{v}}\sqrt{l_{c}^{2}+v^{2}}.$$

In which to acquire v, we need to start an initialization process in advance and keep the focus fix on anywhere in the images during the whole test. The initialization parameter v is defined as

(11) $$v=\sum_{i=1}^{N}W_{i}\cdot \sqrt{{\left(\frac{r_{i_{0}}\cdot n_{v_{i0}}}{h_{r_{i}}\cdot re}\right)}^{2}-l_{c_{i_{0}}}^{2}},$$

where N is number of detected users in an image, $W_{i}$ is the weight of user i, $r_{i_{0}}$ denotes known initial relative range between user i and the camera, $n_{v_{i0}}$, $l_{c_{i_{0}}}$ are initial measurements of value $n_{v}$ and $l_{c}$ of user i respectively.$h_{r_{i}}$ is user i’s height. In this initial process for determining image distance of a camera, commonly we can get an image of multi-users captured by the camera. With knowledge of the initial range from each user to the camera, we can get a group of image distance values. With us of $W_{i}$, a mean value of this group is regarded as the final image distance for this camera.

In addition, a proper camera calibration is needed to use the above pin-hole camera model for resolving ranging and angulation issue. Removal of lens’ distortion from the images and principal point correction are the main tasks in camera calibration. Here, we use a typical camera calibration method by Zhengyou Zhang [59] to obtain intrinsic, lens distortion parameters of the test camera beforehand.

#### 3.2.2. Angulation Model

**Figure 4:**
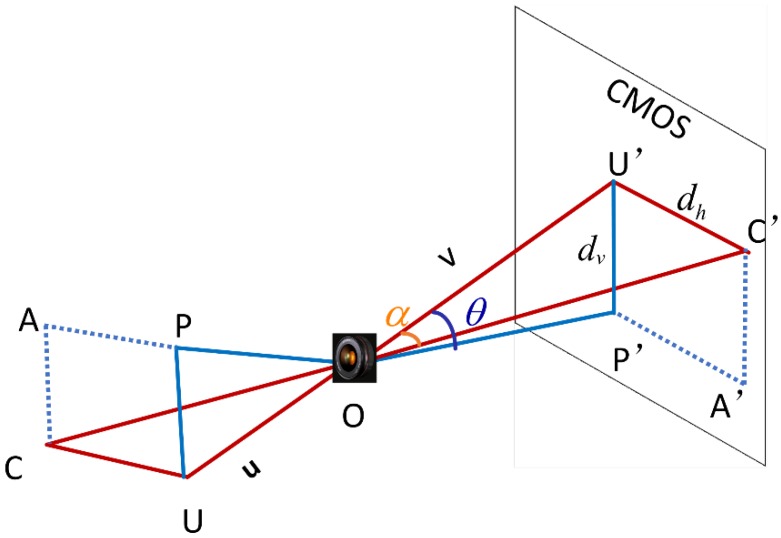
The monocular vision-based angulation model.

The proposed angulation model is illustrated in Figure 4. The projections of a point A onto the horizontal plane that passing through the optical axis and onto its perpendicular plane are points C and P, respectively, and projections of them onto the image plane are points $C^{\prime }$ and $P^{\prime }$.

The azimuth and elevation of A in the observer’s local system are defined as

(12) $$\alpha_{im}=\arctan\frac{d_{h}}{v},$$

(13) $$\theta_{im}=\arctan\frac{d_{v}}{v},$$

where azimuth angle is assumed to be positive by turning clockwise, and elevation angle is assumed to be positive when target lies over the horizontal plane.

### 3.3. IMU/MVRM Integrated System

Considering a IMU/MVRM integrated system which comprises a group of users $U=\left[u_{i}|i\in K\right]$ and a group of cameras $C=\{C_{u_{i}}(t)|i\in K\}$, K={1,⋯,k} denotes a set of users’ ID, $C_{u_{i}}(t)$ is considered to be a set of cameras which can observe user $u_{i}$ at current time t. Each camera is considered to be an anchor, and every single user is equipped with a 6DOF IMU combining a 3-axis gyroscope and a 3-axis accelerometer. The focus in this section is to create dynamical and observation models of the integrated system in accordance with the framework of the extended Kalman filter.

#### 3.3.1. Dynamical Model

The commonly used IMU sensor error models are written as

(14) $$\delta \dot{\rho }=-\omega_{en}\times \delta \rho +\delta v,$$

(15) $$\delta \dot{v}=-(2\omega_{ie}+\omega_{en})\times \delta v-\delta \psi \times f+\delta f^{b},$$

(16) $$\delta \dot{\psi }=-(\omega_{ie}+\omega_{en})\times \delta \psi +\delta \omega_{ib}^{b},$$

where δρ, δv, δφ refer to position, velocity and attitude error vectors expressed in east-north-up coordinates system (ENU) respectively.$\omega_{\mathrm{en}}$ indicates angular rate of navigation frame related to earth; $\omega_{\mathrm{ie}}$ is the earth’s angular rate, and f is specific force.$\delta \omega_{ib}^{b}$, $\delta f^{b}$ are gyro drifts and accelerometer biases, respectively.

For any single user $u_{i}$, a 15-dimension state vector is defined as:

(17) $$x_{u_{i}}={\left[\delta \rho_{u_{i}},\delta v_{u_{i}}^{n},\varphi_{u_{i}},\delta \omega_{u_{i}}^{b},\delta f_{u_{i}}^{b}\right]}^{T}.$$

The dynamical model is expressed as:

(18) $${\dot{x}}_{u_{i}}=F_{u_{i}}\cdot x_{u_{i}}+G_{u_{i}}\cdot w_{u_{i}},$$

where $F_{u_{i}}$ is state transition model, $G_{u_{i}}$ is control model, $w_{u_{i}}$ is the process noise vector which is assumed to be drawn from a zero mean Gaussian normal distribution.

#### 3.3.2. Observation Model

The measurements of the integrated system comprise ranges and angles of users with respect to their visible anchors. First, a simple model involved in a single user with an anchor is created.

The ranging function $d_{u_{i}c_{j}}$ and angulation function $a_{{}_{u_{i}c_{j}}}$ of user $u_{i}$ related to anchor $c_{j}$ are:

(19) $$d_{u_{i}c_{j}}=\sqrt{\Delta e_{ij}{}^{2}+\Delta n_{ij}{}^{2}+\Delta u_{ij}{}^{2}}+v_{u_{i}c_{j}},$$

(20) $$a_{{}_{u_{i}c_{j}}}=\left[\begin{array}{c} \alpha_{u_{i}c_{j}}\\ \theta_{u_{i}c_{j}} \end{array}\right]=\left[\begin{array}{c} \arctan\frac{\Delta e_{ij}}{\Delta n_{ij}}+n_{u_{i}c_{j}}\\ \arcsin(\frac{\Delta u_{ij}}{\sqrt{\Delta e_{ij}^{2}+\Delta n_{ij}^{2}+\Delta u_{ij}^{2}}})+m_{u_{i}c_{j}} \end{array}\right],$$

where $v_{u_{i}c_{j}}$, $n_{u_{i}c_{j}}$ and $m_{u_{i}c_{j}}$ are observation noises which are assumed to be drawn from a zero mean Gaussian normal distribution. $\Delta p={\left[\Delta e_{ij},\Delta n_{ij},\Delta u_{ij}\right]}^{T}$ is difference of positions of user $u_{i}$ with respect to anchor $c_{j}$ in ENU coordinates.

The observation model of $u_{i}$ with $c_{j}$ is made by:

(21) $$z_{u_{i}c_{j}}=[\begin{array}{c} d_{{}_{u_{i}c_{j}}}^{MVRM}-d_{{}_{u_{i}c_{j}}}^{IMU}\\ a_{u_{i}c_{j}}^{MVRM}-a_{u_{i}c_{j}}^{IMU} \end{array}],$$

where $d_{{}_{u_{i}c_{j}}}^{MVRM}$, $a_{u_{i}c_{j}}^{MVRM}$ are ranges and angles measured by using MVRM method, $d_{{}_{u_{i}c_{j}}}^{IMU}$, $a_{u_{i}c_{j}}^{IMU}$ are ranges and angles predicted by user’s IMU.

Extend the measuring function of a user and an anchor to its function with multiple anchors. Assuming the set of *N* cameras which can observe user $u_{i}$ simultaneously is $C_{u_{i}}=\{c_{j}|j∍\{1,\cdots ,N\}\}$, and the observation model extended to

(22) $$z_{u_{i}C_{u_{i}}}=[\begin{array}{c} z_{u_{i}c_{1}}\\ \vdots \\ z_{u_{i}c_{n}} \end{array}]=H_{u_{i}C_{u_{i}}}\cdot x_{u_{i}}+v_{u_{i}},$$

with

(23) $$H_{u_{i}C_{u_{i}}}=\left[\begin{array}{c} \frac{\partial d_{u_{i}c_{1}}}{\partial x_{u_{i}}}\\ \frac{\partial a_{u_{i}c_{1}}}{\partial x_{u_{i}}}\\ \vdots \\ \frac{\partial d_{u_{i}c_{N}}}{\partial x_{u_{i}}}\\ \frac{\partial a_{u_{i}c_{N}}}{\partial x_{u_{i}}} \end{array}\right].$$

Extend the dynamical and observation models applied to an IMU/MVRM integrated system comprises a group of users $U=\left[u_{i}|i\in K\right]$ and a group of cameras $C=\{C_{u_{i}}(t)|i\in K\}$ to

(24) $$X={\left[x_{u_{1}},\cdots x_{u_{k}}\right]}^{T},$$

(25) $$F={\left[F_{u_{1}},\cdots F_{u_{k}}\right]}^{T},$$

(26) $$Z={\left[z_{u_{1}C_{u_{{}_{1}}}},\cdots ,z_{u_{k}C_{u_{{}_{k}}}}\right]}^{T},$$

(27) $$H={\left[H_{u_{1}C_{u_{{}_{1}}}},\cdots ,H_{u_{k}C_{u_{{}_{1}}}}\right]}^{T},$$

## 4. Tests and Results

### 4.1. Experiments Preparations

To test the performance of the proposed approach, several tests were conducted at Northwestern Polytechnical University. Before tests work, we performed several tasks for test preparations. The test area of the rectangle path with 23 markers in an office is 4.2 m × 2.4 m.

First, as discussed in Section 2, an offline training to build a Faster R-CNN by using a Caffe library was conducted. We randomly took and labeled 200 images of a test person in the test field to create a dataset according with PASCAL VOC 2007. This new dataset consisted of a test dataset with 80 images, training dataset with 72 images and validation dataset with 80 images. Next, perform the camera calibration to extract intrinsic, lens distortion parameters of the camera with a 90 cm × 115 cm checkerboard pattern by using Camera Calibration Toolbox for MATLAB. Finally, perform the initialization for each camera to determine an initial image distance.

### 4.2. Performance Evaluation

#### 4.2.1. The Fine-Tuned Faster R-CNN

**Figure 5:**
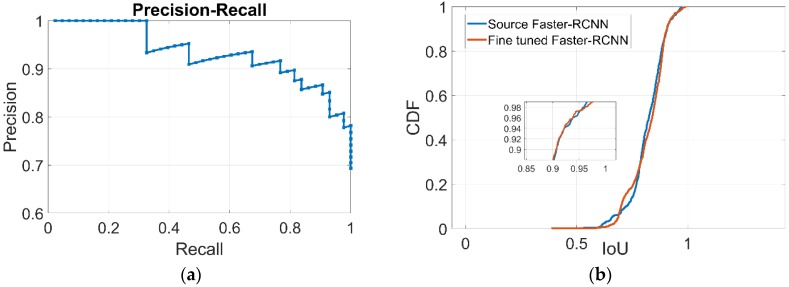
The performance of the fine-tuned Faster R-CNN. (**a**) The Precision-Recall Curve; (**b**) The cumulative probability distribution (CDF) of IoU.

As discussed in Section 3.1, the precision, recall rate and IoU are used to evaluate the accuracy of detection and location. To test the performance of the fine-tuned Faster R-CNN, we chose 80 images to create a dataset which included 45 positive samples and 35 negative samples. The precision-recall curve is shown in Figure 5a. The precision is reaching approximately 90% with a recall rate of 80%. The mAP for the fine-tuned Faster R-CNN is increased to 93.5%, compared to 58.7% for the source Faster-RCNN on the PASCAL 2007. Figure 5b shows the cumulative distribution functions plotted for IoU. The averages of IoU for the fine-tuned and source Faster R-CNN are approximately 0.823 and 0.819, respectively. In summary, the fine-tuned Faster R-CNN is more applicable to the detections on our target task which has an improved detection accuracy.

#### 4.2.2. Analysis of Ranging and Angulation Model in Obstacle-Free Environments

**Figure 6:**
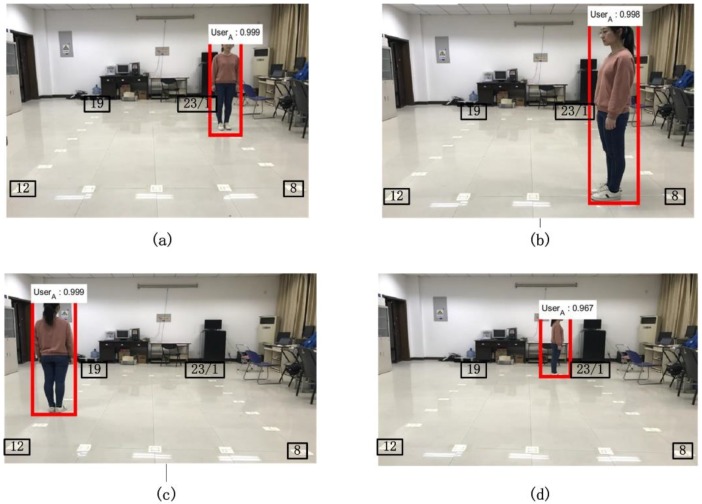
A person captured in four phases. (**a**) phase 1, marker 1–8th; (**b**) phase 2, marker 9–12th; (**c**) phase 3, marker 13–19th; (**d**) phase 4, marker 20–23th.

Highly precise measurements including ranges and angles are vital to correct IMU’s drifts and determine positions and headings of moving users. In this section, we test the performance of the proposed ranging and angulation model, and investigate how the location deviation of the detected bounding box obtained by Faster R-CNN affects the measuring accuracy. We took respectively 40 images of a test person locating at 23 markers with a stationary camera, and categorized markers into four phases by diverse angles and distances with respect to the camera. The four phases are detailed in Figure 6.

**Figure 7:**
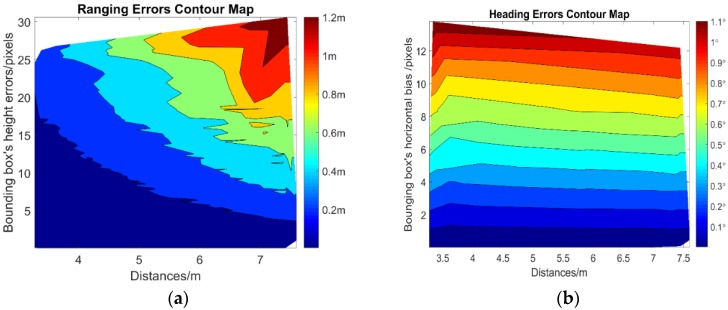
The contour map of measurements errors. (**a**) Ranging errors; (**b**) angulation errors.

As we can see from Figure 7a, the ranging accuracy based on the proposed ranging model is affected by detected bounding box’s height errors and diverse measuring distances, and bounding box’s height errors decrease the ranging accuracy dramatically when measure the target at a long distance. For example, for the bounding box’s height errors at 25 pixels, the ranging error is 0.3 m when measuring the target less than 4 m, but when greater than 7 m, the ranging error is rapidly increased to 1.2 m. Figure 7b shows that the heading errors primarily depend on the bounding box’s horizontal biases. Unlike the analysis of the ranging accuracy, the heading accuracy has no bearing on the measuring distances. For a maximum of image center horizontal biases at 14 pixels, the heading error reaches approximately 1.1°.

**Figure 8:**
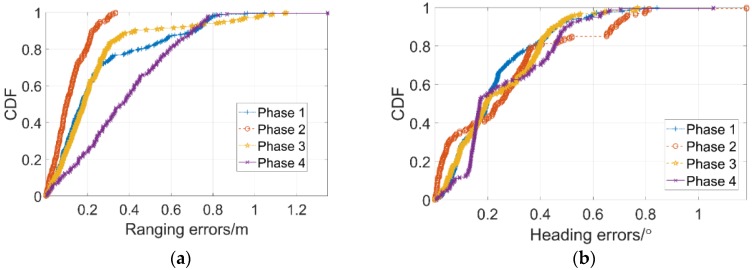
The cumulative distribution functions of errors for four phases. (**a**) CDF of ranging errors; (**b**) CDF of angulation errors.

Figure 8 shows the cumulative distribution functions of errors for four phases. Table 1 summarizes the errors in four phases. P2 shows the preferable ranging performance in phase 2 due to the shortest measure distances compared to the results of other phases, however, it appears no obvious distinctions of heading errors among four phases which is indeed proved to be consistent with the analysis of results in Figure 7. Due to the symmetric properties of markers’ locations between P1 and P3, the CDF in Figure 8 and estimated position error data of P1 and P3 in Table 1 both show more similar results.

Table 1 shows the estimated position error respectively caused by ranging error and by heading error in four phases. It is shown that the RMSEs in estimated position values respectively caused by ranging and heading differ by one order of magnitude, and the effect on position errors caused by heading is less than by ranging.

From the above tests, we conclude that the ranging accuracy is affected by both bounding box’s height errors and diverse measuring distances, however, the heading accuracy is solely interfered with bounding box’s horizontal biases. In addition, the inaccuracy in the vision part may come from human walking posture. For example, pedestrian’s spine is curved too far forwards or backwards and greater stride variation when walking that will make pedestrian’s height errors in images. This fact that the difference between detected pedestrian’s height and the true height caused by walking posture somehow influences the distance values. In this paper, we neglect this inaccuracy.

#### 4.2.3. Analysis of Ranging and Angulation Model in Obstacle Environments

**Figure 9:**
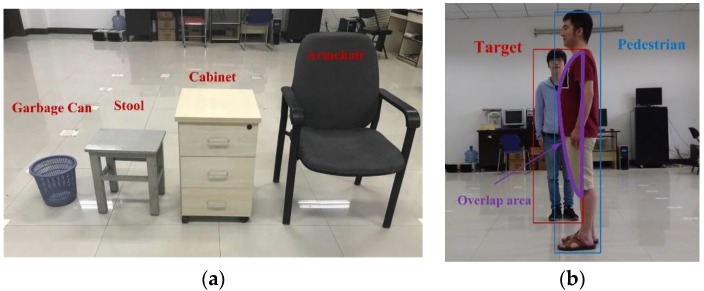
Indoor obstacles environments. (**a**) Stationary obstacles; (**b**) pedestrian blockage.

In general, indoor objects, such as furniture and pedestrians, occasionally block cameras from viewing the moving users. In this section, we investigate the measuring performance of the proposed MVRM method when stationary obstacles or pedestrians block the camera from observing the moving users in indoor environment. As discussed above, two cases that analyze effect of obstructions by various stationary obstacles and a pedestrian on bounding box’s height and bounding box’s horizontal biases at a same measuring distance are considered below. In Figure 9a, common obstacles to be used in the test field which primary cause bounding box’s height errors by blocking the lower half of the user’s body. Figure 9b shows a scenario in which the pedestrian blocks a little view of camera from viewing the user. This scenario causes both bounding box’s height errors and horizontal biases. In this test, the camera is perpendicularly placed at the location with a height of 1.2 m and a distance from the target of 5.1 m. Each obstacle all located at the same location in front of the target.

##### Case 1: Stationary Obstacles

According to dimensions of common obstacles in indoor environments, an armchair, cabinet, stool, and garbage can are used to test the ranging and heading accuracy.

**Figure 10:**
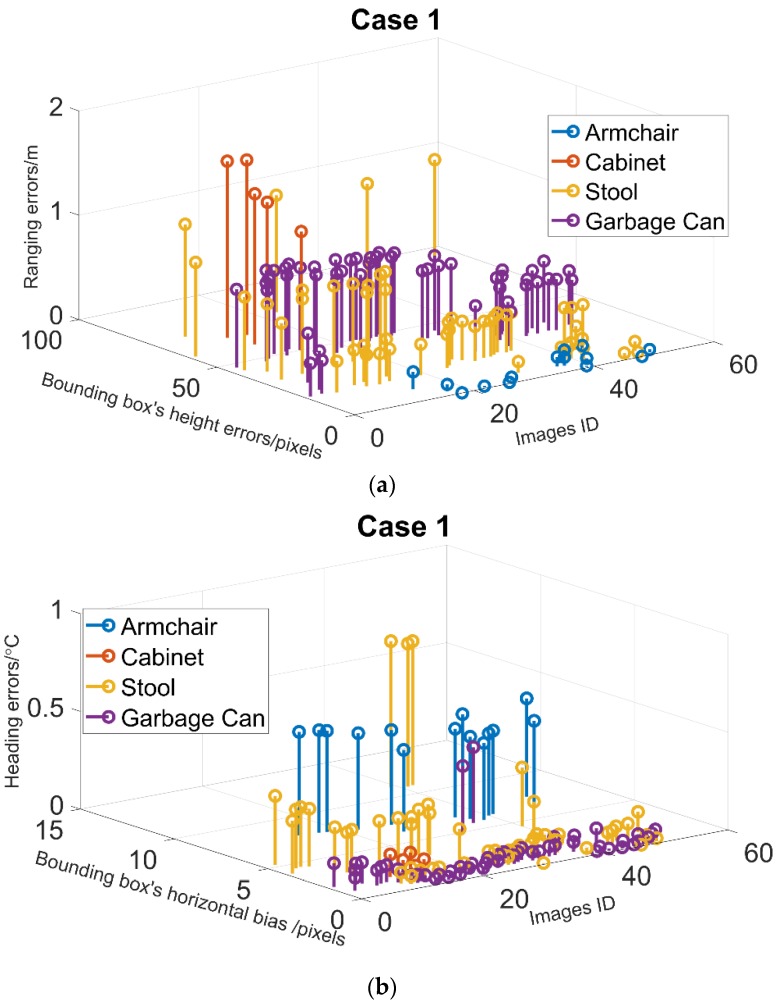
The effect of obstruction by various stationary obstacles. (**a**) Ranging error; (**b**) angulation error.

Figure 10a shows that greater errors in bounding box’s height most likely induce greater ranging errors at a same measuring distance, which is consistent with the analysis of results in Figure 7a. Generally, the overlap area of the target with obstacles depends on the position and attitude of camera respective to the target and its obstacles. In our test condition, obstruction by the armchair yielding the least overlap area of the lower half of the body, which leads to a minimal RMSE of ranging as 0.1 m. Garbage can blockage scenario is second, followed by stool blockage scenario, and the obstruction by the cabinet leads to the greatest RMSE as 1.5 m.

Figure 10b shows the similar results that greater bounding box’s horizontal biases most likely induce greater heading errors in Figure 7b. The minimum RMSE of heading is caused by the occlusion by the narrowest garbage at 0.1°, however, the maximum’s is caused by the occlusion by the widest armchair at 0.47°.

In the view of the above tests, stationary obstacles used in these tests have greater influences on ranging accuracy than heading accuracy. The detection failure rates caused by the cabinet and armchair in blockage scenarios are 90% and 72% respectively, which is shown in Table 2.

##### Case 2: Pedestrian Blockage

**Figure 11:**
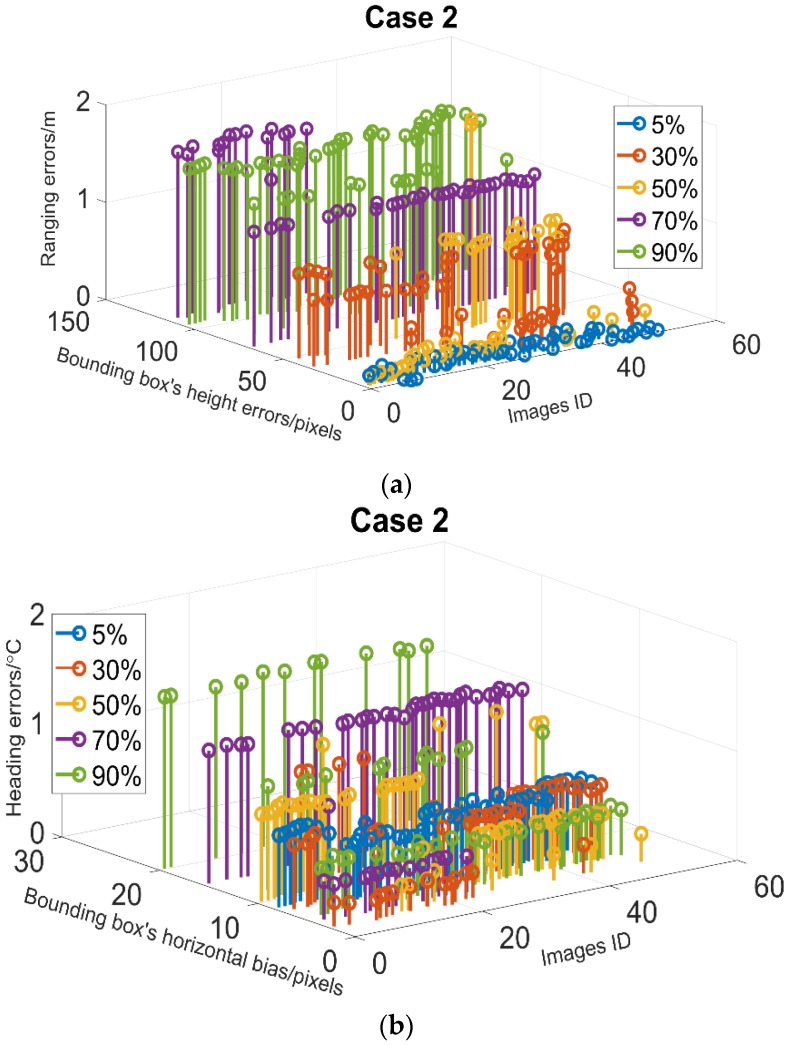
The effect of obstruction under five overlap ratios of target and the pedestrian. (**a**) Ranging error; (**b**) angulation error.

In this section, we investigate the potential effect of obstructions by an unidentified pedestrian by our vision system on ranging and heading accuracy. The test results under 5 various overlap ratios of the target and the pedestrian are presented in Figure 11.

As shown in Figure 11a,b, greater overlap ratios generally create greater bounding box’s height errors and imaging horizontal biases which both decrease the accuracy of the ranging and heading. In general, when the overlap ratio approaching 50%, the ranging errors are greater than 1 m, while the heading errors are slightly greater than 1°. In terms of the detection failure rates, unlike the results in case 1, the detection ability is insensitive to the blocking by a pedestrian.

From the results in Table 2 and the above tests, the blockage cases more significantly decrease the accuracy of ranging than that of heading in our tests. Specifically, the effect of a pedestrian on the heading errors is greater than that of stationary obstacles. However, the possibility of detection failure caused by stationary obstacles is much higher than the pedestrian case.

#### 4.2.4. Positioning Results and Analysis

**Figure 12:**
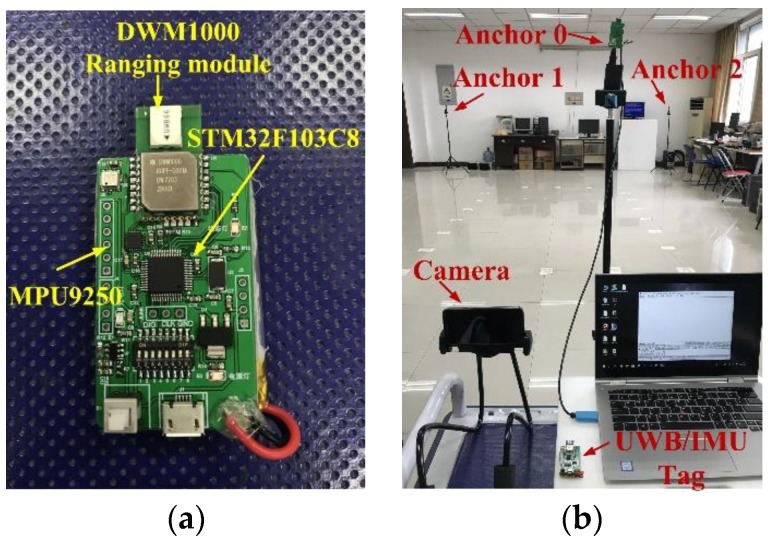
The setup for the experiment in test field. (**a**) The carried tag; (**b**) the setup for the test indoor positioning system.

To further evaluate the positioning ability of the proposed integrated IMU/MVRM approach, a test for a single user and an external camera is implemented in obstacles environment. The setup for this experiment is shown in Figure 12. The used tag includes one IMU (MPU9250) and one UWB module (DWM1000) and build a UWB positing system with three anchors for contrast deployed in the test field. The methods run on a laptop with an Intel Core i5-3230M CPU, operating at 2.4 GHz, and 4 GB of RAM. In order to time-synchronize the IMU readings with the camera frame, we processed each image frame from video file individually and tag the precise time stamp in millisecond-lever with OpenCV. The IMU data is being logged with millisecond-lever precision and interpolate the IMU readings by time to match camera frame time.

**Figure 13:**
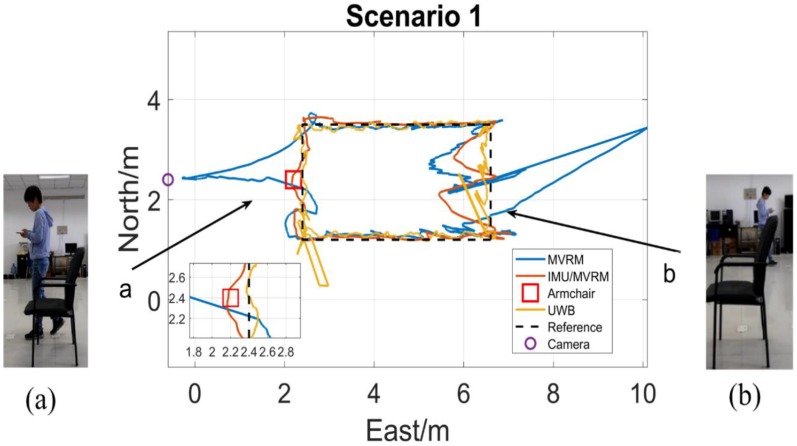
The horizontal trajectory in armchair scenario. (**a**) The armchair blocked the view of the camera from seeing the test person at the marker 10th, and failed to detect the person; (**b**) the armchair blocked the view of the camera from seeing the test person near the marker 22nd, and failed to detect the person.

**Figure 14:**
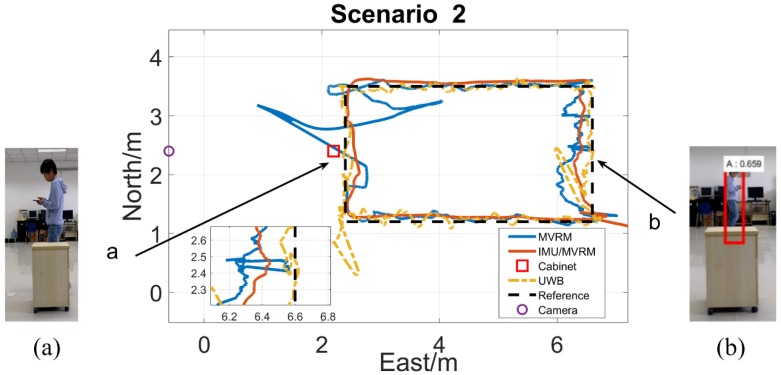
The horizontal trajectory in cabinet scenario. (**a**) The cabinet blocked the view of the camera from seeing the test person at the marker 10th, and failed to detect the person; (**b**) the cabinet blocked the little view of the camera from seeing the test person near the marker 21st, and succeed to detect but caused image height errors.

**Figure 15:**
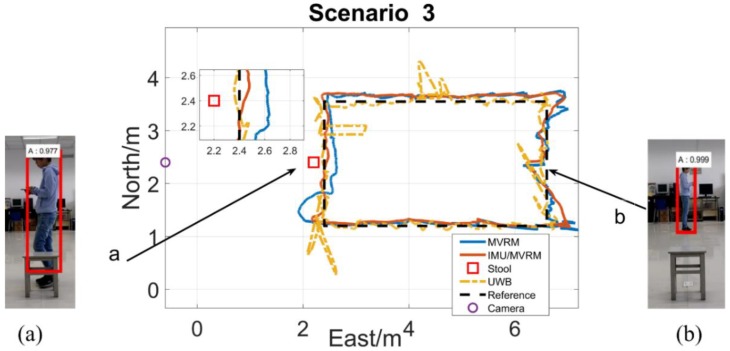
The horizontal trajectory in stool scenario. (**a**) the stool blocked the little view of the camera from seeing the test person at the marker 10th, succeed to detect but caused image height errors; (**b**) the stool is not able to block any view of the camera from seeing the test person near the marker 21st, succeed to precisely detect.

**Figure 16:**
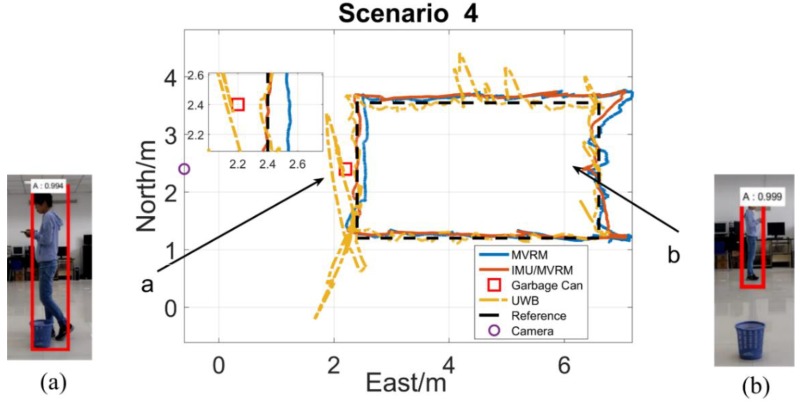
The horizontal trajectory in garbage can scenario. (**a**) The garbage can blocked the little view of the camera from seeing the test person at the marker 10th, and succeed to detect but caused little image height errors; (**b**) the stool is not able to block any view of the camera from seeing the test person near the marker 21st, and succeed to precisely detect.

**Figure 17:**
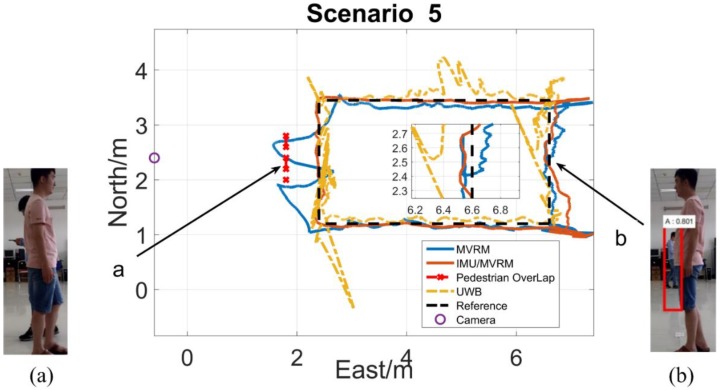
The horizontal trajectory in pedestrian scenario. (**a**) The pedestrian blocked the view of the camera from seeing the test person at the marker 10th and failed to detect the person; (**b**) the pedestrian blocked little view of the camera from seeing the test person near the marker 21st, and succeed to detect but caused larger image height errors.

The test person carried the tag walking along the rectangle path with a normal speed. The two-dimensional local coordinate system is built in the test field. The initial position of the test person is (6.6, 1.2), and the external camera is (−0.6, 2.4), each obstacle all locates at (2.2, 2.4) which is near the marker 10th. Figure 13, Figure 14, Figure 15, Figure 16 and Figure 17 are horizontal positioning trajectories by using the MVRM, IMU/MVRM, and UWB in 5 scenarios. Table 3 summarizes the positioning errors.

As observed from Figure 13, the position estimations by using the MVRM method appears as a number of strong outliers continually occurring in blockage phase (a) due to lacking visual measurements including ranges and headings. Unfortunately, in blockage phase (b), the high armchair still blocks the camera from acquiring visual measurements which causes a large number of strong outliers in positioning trajectory again. For the fused IMU/MVRM solution, the position estimations predicted by IMU during blockage phase (a) and (b) which effectively reduces and removes the errors in positioning trajectory. For the UWB, in the dense multipath environments, such as corner, the positioning results are not satisfying.

The positioning results by using MVRM method in Figure 14a are similar to that in scenario 1(a), while the slightly narrow cabinet only causing lacking a small amount of visual measurement in shorter period, which suffers from smaller values of outliers in positioning trajectory compared with that in scenario 1(a). For the Figure 14b, the shorter cabinet rarely interferences with the person detection in images, however, it brings out bounding box’s height errors. These visual errors are reduced by using the fused IMU/MVRM and the improved positioning results are achieved. The results of UWB are similar to that in scenario 1.

As shown in Figure 15, for blockage phase (a), the outliers, in this context, are results of the visual measurements acquired by using the MVRM method. These measurements departing from the normal range due to the detected shorter bounding boxes which are affected by the stool. Nevertheless, the fused IMU/MVRM solution can reduce and remove the positioning estimations errors substantially. For (b), the test person is fully visible during this period, therefore, similar results of the two solutions are shown at the east side of the trajectories. In terms of UWB, except for the similarities in scenario 1 and 2, there are greater positioning errors in the north trajectory because of multipath effect from a row of metal cabinets at the north of the test field.

Figure 16 shows the similar results with scenario 3. The garbage can has the smallest dimension, therefore, the MVRM solution which causes the smallest positioning estimations errors among all blockage scenarios shows slightly unsmooth trajectories in phase (a) compared with the fused IMU/MVRM solution. The UWB solution are similar to that in scenario 3.

In Figure 17, for (a) and (b), the positioning results by using the MVRM solution shows greater errors due to lacking visual measurements affected by the pedestrian during these two blockage periods. Similarly, the fused IMU/MVRM solution significantly decreases the positioning errors by integrating with the position estimations predicted by IMU during blockage periods. The results of using UWB are similar to that in scenario 3 as well. Specifically, the UWB solution shows poor performance because of the effect of the pedestrian in phase (a).

In summary, the pure MVRM solution is more sensitive to the effect of both stationary obstacles and pedestrians, however, the robust fused IMU/MVRM solution integrating with the IMU’s predications and visual measurements which can effectively and significantly decrease the positioning errors in short period. Indoor objects commonly easily lead to the multipath effect. In dense multipath environments, such as corner, the UWB solution shows poor performance compared with the proposed IMU/MVRM. Therefore, this fused IMU/MVRM solution is more applicable to the dense multipath scenarios where the obstacles may partially block the view of the cameras.

## 5. Conclusions and Future Work

A novel fusion of IMU with visual measurements acquired by cameras to determine robust and accurate poses of the test person for indoor positioning is presented. The visual measurements including ranges and angles are obtained by the proposed MVRM method with the assistant of a fine-tuned Faster R-CNN which is used to detect and locate the target in images captured by the cameras. We developed an extended Kalman filter for integrating IMU data with ranges and angles to obtain a more robust and accurate estimations. We designed several tests to evaluate the performance. The results experimentally show that the ranging accuracy is affected by both bounding box’s height errors and diverse measuring distances, however, the heading accuracy is solely interfered with bounding box’s horizontal biases. The blockage cases more significantly decrease the accuracy of ranging than that of heading in our tests. The positioning experiments for a single user with a camera in five scenarios were implemented in indoor environments. The robust fused IMU/MVRM solution can effectively and significantly decrease the positioning errors and shows better performance in the dense multipath scenarios compared with the pure MVRM and UWB solution.

We think that the proposed approach for indoor positioning can be applied in current location-based applications in buildings where equipped IP cameras in near future. To perfectly implement it in real applications, developing a more fast and accurate detection in more complicated indoor environments is a critical task, and acquiring the more accurate poses of installed cameras is able to further enhance the performance of the proposed approach. In our proposed framework, the position of each camera is independent. We will consider a multi-camera model with known relative pose to improve the results in the future work.

## Figures and Tables

**Table 1:** The estimated position error caused by ranging and heading error in four phases.

Phases		P1	P2	P3	P4
**By Ranging/m**	**RMSE**	0.33	0.14	0.29	0.42
	**STD**	0.26	0.1	0.28	0.39
	**Max.**	1.01	0.33	1.07	1.35
**By Heading/m**	**RMSE**	0.03	0.02	0.03	0.04
	**STD**	0.01	0.01	0.02	0.04
	**Max.**	0.07	0.07	0.09	0.14

**Table 2:** The ranging and heading error and detection failure rates in obstacle environments.

Blockage Categories		Armchair	Cabinet	Stool	Garbage	Pedestrian (5%)	Pedestrian (30%)	Pedestrian (50%)	Pedestrian (70%)	Pedestrian (90%)
**Range/m**	**RMSE**	0.08	1.45	0.53	0.64	0.1	0.61	0.59	1.36	1.53
	**STD**	0.08	0.23	0.52	0.15	0.09	0.34	0.5	0.11	0.24
	**Max.**	0.17	1.7	1.12	0.82	0.15	0.88	1.57	1.73	1.62
**Heading** /°	**RMSE**	0.46	0.11	0.24	0.1	0.59	0.54	0.65	0.91	1
	**STD**	0.05	0.02	0.24	0.09	0.05	0.4	0.41	0.48	0.42
	**Max.**	0.5	0.13	0.73	0.12	0.7	1	1.09	1.25	1.6
**Detection Failure rates**		72%	90%	0	0	0	0	0	0	8%

**Table 3:** The horizontal positioning errors.

Horizontal Errors/m		Scenario 1		Scenario 2		Scenario 3		Scenario 4		Scenario 5	
		East	North	East	North	East	North	East	North	East	North
**UWB**	**RMSE**	0.16	0.12	0.2	0.1	0.35	0.14	0.13	0.33	0.15	0.4
	**STD**	0.09	0.05	0.1	0.04	0.32	0.11	0.1	0.22	0.1	0.2
	**Max.**	0.58	0.92	0.22	0.89	0.22	0.88	0.72	1.38	0.62	1.52
**MVRM**	**RMSE**	0.76	0.2	0.51	0.11	0.2	0.06	0.19	0.08	0.35	0.18
	**STD**	0.1	0.02	0.11	0.01	0.08	0.02	0.1	0.02	0.13	0.02
	**Max.**	3.56	0.92	1.49	0.77	0.6	0.11	0.59	0.13	3.7	0.17
**IMU/MVRM**	**RMSE**	0.24	0.07	0.12	0.07	0.2	0.06	0.18	0.08	0.17	0.12
	**STD**	0.08	0.01	0.07	0.01	0.04	0.01	0.05	0.02	0.08	0.03
	**Max.**	0.99	0.23	0.59	0.08	0.54	0.14	0.46	0.13	0.81	0.25

## References

[1] De Angelis G., Pasku V., De Angelis A., Dionigi M., Mongiardo M., Moschitta A., Carbone P. (2015). An indoor AC magnetic positioning system. IEEE Trans. Instrum. Meas..

[2] Shi G.W., Ming Y. (2016). Survey of indoor positioning systems based on ultra-wideband (UWB) technology. Wireless Communications, Networking and Applications.

[3] Brena R.F., Garcia-Vazquez J.P., Galvan-Tejada C.E., Munoz-Rodriguez D., Vargas-Rosales C., Fangmeyer J. (2017). Evolution of indoor positioning technologies: A survey. J. Sens..

[4] Molina B., Olivares E., Palau C.E., Esteve M. (2018). A multimodal fingerprint-based indoor positioning system for airports. IEEE Access.

[5] Hwang I., Jang Y.J. (2017). Process mining to discover shoppers’ pathways at a fashion retail store using a WiFi-base indoor positioning system. IEEE Trans. Autom. Sci. Eng..

[6] Mashuk M.S., Pinchin J., Siebers P.O., Moore T. A smart phone based multi-floor indoor positioning system for occupancy detection. Proceedings of the 2018 IEEE/ION Position, Location and Navigation Symposium (PLANS).

[7] Van der Ham M.F.S., Zlatanova S., Verbree E., Voute R. Real time localization of assets in hospitals using quuppa indoor positioning technology. Proceedings of the First International Conference on Smart Data and Smart Cities (30th UDMS).

[8] Zhuang Y., Syed Z., Li Y., El-Sheimy N. (2016). Evaluation of two WiFi positioning systems based on autonomous crowdsourcing of handheld devices for indoor navigation. IEEE Trans. Mob. Comput..

[9] Faragher R., Harle R. An analysis of the accuracy of Bluetooth low energy for indoor positioning applications. Proceedings of the 27th International Technical Meeting of the Satellite Division of the Institute of Navigation (ION GNSS 2014).

[10] Lin X.Y., Ho T.W., Fang C.C., Yen Z.S., Yang B.D., Lai F.P. A mobile indoor positioning system based on iBeacon technology. Proceedings of the 2015 37th Annual International Conference of the IEEE Engineering in Medicine and Biology Society (EMBC).

[11] Huang C.H., Lee L.H., Ho C.C., Wu L.L., Lai Z.H. (2015). Real-time RFID indoor positioning system based on Kalman-filter drift removal and Heron-bilateration location estimation. IEEE Trans. Instrum. Meas..

[12] Yang Z.X., Zhang P.B., Chen L. (2016). RFID-enabled indoor positioning method for a real-time manufacturing execution system using OS-ELM. Neurocomputing.

[13] Yang D., Xu B., Rao K.Y., Sheng W.H. (2018). Passive infrared (PIR)-based indoor position tracking for smart homes using accessibility maps and a-star algorithm. Sensors.

[14] Wu C.X., Mu Q., Zhang Z.B., Jin Y.F., Wang Z.Y., Shi G.Y. Indoor positioning system based on inertial mems sensors: Design and realization. Proceedings of the 2016 IEEE International Conference on Cyber Technology in Automation, Control, and Intelligent Systems (CYBER).

[15] Kasmi Z., Norrdine A., Blankenbach J. (2015). Towards a decentralized magnetic indoor positioning system. Sensors.

[16] Alarifi A., Al-Salman A., Alsaleh M., Alnafessah A., Al-Hadhrami S., Al-Ammar M.A., Al-Khalifa H.S. (2016). Ultra-wideband indoor positioning technologies: Analysis and recent advances. Sensors.

[17] Mazhar F., Khan M.G., Sallberg B. (2017). Precise indoor positioning using UWB: A review of methods, algorithms and implementations. Wirel. Pers. Commun..

[18] Feng C., Au W.S.A., Valaee S., Tan Z.H. (2012). Received-signal-strength-based indoor positioning using compressive sensing. IEEE Trans. Mob. Comput..

[19] Janicka J., Rapinski J. Application of RSSI based navigation in indoor positioning. Proceedings of the 2016 Baltic Geodetic Congress (Bgc Geomatics).

[20] Xia S.X., Liu Y., Yuan G., Zhu M.J., Wang Z.H. (2017). Indoor fingerprint positioning based on Wi-Fi: An overview. ISPRS Int. J. Geo-Inf..

[21] Tang J., Chen Y.W., Chen L., Liu J.B., Hyyppa J., Kukko A., Kaartinen H., Hyyppa H., Chen R.Z. (2015). Fast fingerprint database maintenance for indoor positioning based on UGV SLAM. Sensors.

[22] Alsudani A. (2018). NLOS mitigation and ranging accuracy for building indoor positioning system in UWB using commercial radio modules. AIP Conf. Proc..

[23] Harle R. (2013). A survey of indoor inertial positioning systems for pedestrians. IEEE Commun. Surv. Tutor..

[24] Correa A., Barcelo M., Morell A., Vicario J.L. (2017). A review of pedestrian indoor positioning systems for mass market applications. Sensors.

[25] Li X., Wang J., Liu C.Y. (2015). A Bluetooth/PDR integration algorithm for an indoor positioning system. Sensors.

[26] Tian Z.S., Fang X., Zhou M., Li L.X. (2015). Smartphone-based indoor integrated WiFi/MEMS positioning algorithm in a multi-floor environment. Micromachines.

[27] Kok M., Hol J.D., Schon T.B. (2015). Indoor positioning using ultra-wideband and inertial measurements. IEEE Trans. Veh. Technol..

[28] Anup S., Goel A., Padmanabhan S. Visual positioning system for automated indoor/outdoor navigation. Proceedings of the TENCON 2017—2017 IEEE Region 10 Conference.

[29] Huang Z., Zhu J.G., Yang L.H., Xue B., Wu J., Zhao Z.Y. (2015). Accurate 3-D position and orientation method for indoor mobile robot navigation based on photoelectric scanning. IEEE Trans. Instrum. Meas..

[30] Endo Y., Sato K., Yamashita A., Matsubayashi K. Indoor positioning and obstacle detection for visually impaired navigation system based on LSD-SLAM. Proceedings of the 2017 International Conference on Biometrics and Kansei Engineering (ICBAKE).

[31] Saputra M.R.U., Markham A., Trigoni N. (2018). Visual SLAM and structure from motion in dynamic environments: A survey. ACM Comput. Surv..

[32] Gui J.J., Gu D.B., Wang S., Hu H.S. (2015). A review of visual inertial odometry from filtering and optimisation perspectives. Adv. Robot..

[33] Schmid K., Hirschmuller H. Stereo vision and IMU based real-time Ego-motion and depth image computation on a handheld device. Proceedings of the 2013 IEEE International Conference on Robotics and Automation.

[34] Li M.Y., Mourikis A.I. (2013). High-precision, consistent EKF-based visual-inertial odometry. Int. J. Robot. Res..

[35] Leutenegger S., Lynen S., Bosse M., Siegwart R., Furgale P. (2015). Keyframe-based visual-inertial odometry using nonlinear optimization. Int. J. Robot. Res..

[36] Qin T., Li P.L., Shen S.J. (2018). Vins-mono: A robust and versatile monocular visual-inertial state estimator. IEEE Trans. Robot..

[37] Antigny N., Servieres M., Renaudin V. Hybrid visual and inertial position and orientation estimation based on known urban 3D models. Proceedings of the 2016 International Conference on Indoor Positioning and Indoor Navigation (IPIN).

[38] Bertozzi M., Broggi A., Fascioli A., Tibaldi A., Chapuis R., Chausse F. Pedestrian localization and tracking system with Kalman filtering. Proceedings of the 2004 IEEE Intelligent Vehicles Symposium.

[39] Colombo A., Fontanelli D., Macii D., Palopoli L. (2014). Flexible indoor localization and tracking based on a wearable platform and sensor data fusion. IEEE Trans. Instrum. Meas..

[40] Kim D.Y., Song H.Y. (2018). Method of predicting human mobility patterns using deep learning. Neurocomputing.

[41] Liu Z.G., Zhang L.M., Liu Q., Yin Y.F., Cheng L., Zimmermann R. (2017). Fusion of magnetic and visual sensors for indoor localization: Infrastructure-free and more effective. IEEE Trans. Multimed..

[42] Jiao J.C., Li F., Deng Z.L., Ma W.J. (2017). A smartphone camera-based indoor positioning algorithm of crowded scenarios with the assistance of deep CNN. Sensors.

[43] Brunetti A., Buongiorno D., Trotta G.F., Bevilacqua V. (2018). Computer vision and deep learning techniques for pedestrian detection and tracking: A survey. Neurocomputing.

[44] Chen X.G., Wei P.X., Ke W., Ye Q.X., Jiao J.B. (2015). Pedestrian detection with deep convolutional neural network. Computer Vision—ACCV 2014 Workshops, Pt I.

[45] Park E., del Pobil A.P., Kwon S.J. (2018). The role of internet of things (IoT) in smart cities: Technology roadmap-oriented approaches. Sustainability.

[46] Leong C.Y., Perumal T., Yaakob R., Peng K.W. Enhancing indoor positioning service for location based internet of things (IoT) a source selecting approach with error compensation. Proceedings of the 2017 IEEE International Symposium on Consumer Electronics (ISCE).

[47] Fathy Y., Barnaghi P., Tafazolli R. (2018). Large-scale indexing, discovery, and ranking for the internet of things (IoT). ACM Comput. Surv..

[48] Lopes S.I., Vieira J.M.N., Reis J., Albuquerque D., Carvalho N.B. (2015). Accurate smartphone indoor positioning using a WSN infrastructure and non-invasive audio for TDOA estimation. Pervasive Mob. Comput..

[49] Wang H., Wen Y.Y., Zhao D.Z. (2017). Differential barometric-based positioning technique for indoor elevation measurement in IoT medical applications. Technol. Health Care.

[50] Jeong J., Yeon S., Kim T., Lee H., Kim S.M., Kim S.C. (2018). Sala: Smartphone-assisted localization algorithm for positioning indoor IoT devices. Wirel. Netw..

[51] De Angelis G., De Angelis A., Pasku V., Moschitta A., Carbone P. A hybrid outdoor/indoor positioning system for IoT applications. Proceedings of the 2015 IEEE International Symposium on Systems Engineering (ISSE) Proceedings.

[52] Liu R., Yuen C., Do T.-N., Jiao D., Liu X., Tan U.X. (2017). Cooperative relative positioning of mobile users by fusing IMU inertial and UWB ranging information. Proceedings of the 2017 IEEE International Conference on Robotics and Automation (ICRA 2017).

[53] Girshick R., Donahue J., Darrell T., Malik J. Rich feature hierarchies for accurate object detection and semantic segmentation. Proceedings of the 2014 IEEE Conference on Computer Vision and Pattern Recognition (CVPR).

[54] Girshick R. Fast R-CNN. Proceedings of the 2015 IEEE International Conference on Computer Vision (ICCV).

[55] Ren S.Q., He K.M., Girshick R., Sun J. (2017). Faster R-CNN: Towards real-time object detection with region proposal networks. IEEE Trans. Pattern Anal..

[56] Zeiler M.D., Fergus R., Fleet D., Pajdla T., Schiele B., Tuytelaars T. (2014). Visualizing and understanding convolutional networks. Computer Vision—ECCV 2014, Pt I.

[57] Everingham M., Van Gool L., Williams C.K.I., Winn J., Zisserman A. (2007). The Pascal Visual Object Classes Challenge 2007 (VOC 2007) Results. http://host.robots.ox.ac.uk/pascal/VOC/voc2007/results/index.shtml.

[58] The Pascal Visual Object Classes Challenge 2012 (VOC 2012) Development Kit. http://host.robots.ox.ac.uk/pascal/VOC/voc2012/htmldoc/devkit_doc.html#SECTION00044000000000000000.

[59] Zhang Z.Y. (2000). A flexible new technique for camera calibration. IEEE Trans. Pattern Anal..

